# A Retrospective Cohort Evaluation of Left Ventricular Remodeling, Perioperative Complications and Outcome in Medium and Large Size Dogs with Patent Ductus Arteriosus after Percutaneous Closure

**DOI:** 10.3390/vetsci10120669

**Published:** 2023-11-24

**Authors:** Melissa Papa, Lorenzo Scarpellini, Danitza Pradelli, Anna Maria Zanaboni, Alessia Mattia, Elisabetta Boz, Cecilia Rossi, Stefania Signorelli, Viviana Forti, Martina Longobardi, Beatrice Pasquinelli, Maria Celeste Gendusa, Davide Gamba, Claudio Maria Bussadori

**Affiliations:** 1 Clinica Veterinaria Gran Sasso, 20134 Milan, Italy; lorenzoscarpellini@clinicaveterinariagransasso.it (L.S.); alessia.mattia25@gmail.com (A.M.); ceciliarossi@clinicaveterinariagransasso.it (C.R.); stefaniasignorelli@clinicaveterinariagransasso.it (S.S.); viviana.forti@clinicaveterinariagransasso.it (V.F.); martinalongobardi@clinicaveterinariagransasso.it (M.L.); beatricepasquinelli@clinicaveterinariagransasso.it (B.P.); celestegendusa@clinicaveterinariagransasso.it (M.C.G.); davidegamba@clinicaveterinariagransasso.it (D.G.);; 2 Computer Science Department, Università degli Studi di Milano, 20133 Milan, Italy; annamaria.zanaboni@unimi.it; 3 Data Science Research Center DSRC, Università degli Studi di Milano, 20133 Milan, Italy

**Keywords:** congenital heart disease, systolic dysfunction, echocardiography, interventional procedure, canine

## Abstract

**Simple Summary:**

Patent ductus arteriosus (PDA) is a frequent congenital heart defect in dogs. The persistent flow through the vascular communication induces an excess of blood flow to the pulmonary circulation and the left heart chambers. The ductal closure leads to a reduction in the left chamber’s volume. The degree of volume reduction after PDA closure is inferior in large size dogs compared to small breed dogs. The present study evidenced worsening heart remodeling with the deterioration of structural and functional condition in dogs with a higher body weight, a larger dimension of the PDA, a more severe heart enlargement or arrhythmias at presentation. This may be associated with perioperative complications and cardiac death. Therefore, when possible, the therapeutic indication is to perform PDA closure as early as possible, in younger dogs with less heart remodeling and major functional capacity. If patients are presented at an older age, the PDA closure must be performed anyway because the untreated persistence of the pathology can induce complications as congestive heart failure, arrhythmias or sudden death.

**Abstract:**

This retrospective cohort study included one hundred fifty-seven medium and large-size dogs with the aim of evaluating the effect of signalment and echocardiographic features on complications, outcomes and left ventricular modifications before and after patent ductus arteriosus (PDA) closure. The patients were divided in two groups based on the heart remodeling after closure: Group A included dogs that had a reduction in the end-systolic volume index (ESVI) after closure compared to the ESVI measured before; Group B included dogs without a reduction in ESVI after closure. Body weight, minimal ductal diameter (MDD) of PDA, end-diastolic volume index and presence of arrhythmias at presentation were significantly higher in Group B compared to Group A. The shortening fraction and ejection fraction after closure were reduced in both groups, but in Group B there was a major reduction, and the mean values indicated a possible systolic dysfunction. Complications during the procedure and death due to cardiac reasons were greater in Group B compared to Group A. In conclusion, a higher body weight, a larger MDD, a more severe heart enlargement or arrhythmias at presentation increased the risk of developing a worsening structural and functional condition after ductal closure, and this can be associated with perioperative complications and cardiac death.

## 1. Introduction

According to the literature, patent ductus arteriosus (PDA) is one of the most common congenital cardiopathies in both pure and crossbreed dogs. It is frequent in large breeds such as German Shepherds, Newfoundlands and in some studies Dobermanns, in small or medium breeds including Maltese, Poodles, Cavalier King Charles Spaniels, Chihuahuas and Border Collies, and females are more affected than males [1,2,3,4]. 

The ductus arteriosus is an arterial communication between the main pulmonary artery and aorta during fetal life which allows the blood flow to bypass the lungs which are not aerated [1,4,5]. Physiologically, it closes few hours after birth to allow neurohormonal and hemodynamic processes which start with lung expansion and consequently lead to an increase in oxygen tension and inhibit prostaglandin production [4,5,6,7]. Sometimes the closure of the ductus does not happen, therefore there is a consequent persistence of a vascular communication within the left-to-right shunt. In this condition the pathophysiologic picture is influenced by an elevated preload in the left heart chambers, with secondary volume overload in the left ventricle and left ventricular hyperkinesis that would be the expected consequence of the Frank–Starling mechanism; in more severe conditions a dilation of the left atrium can also be evidenced [3,6,7,8,9,10,11]. In untreated patients the persistence of this shunt can lead to clinical signs of left congestive heart failure (CHF), as pulmonary edema, arrhythmias or sudden death [7,10]. Currently, a PDA transcatheter occlusion using the Amplatzer Canine Duct Occluder (ACDO) is one of the most used methods for the closure of the defect with a left-to-right shunt [10,12]. 

In humans and dogs, the closure of the PDA is followed by a decreased preload and an increased afterload, with a reduction in the left chamber’s volume and a possible contractility reduction [6,8,9,10,13,14,15]. In dogs, a different response after ductal closure depending on body size has been evidenced: in particular, in small breed dogs there is a greater reduction in indexed ventricular diameters compared to large breeds, and large breed dogs have significantly increased LV dimensions and decreased shortening fractions during follow-up in comparison to mixed and small breed dogs [10,16]. In human medicine, the dimension of the minimal ductal diameter (MDD) of the PDA seems to influence the heart systolic performance after ductal closure [9].

Furthermore, a correlation between age and cardiac remodeling after PDA occlusion in dogs is debated [16,17,18].

The aim of this study was the evaluation of the degree of left ventricle (LV) remodeling in medium and large size dogs after PDA closure, the analysis of the possible factors that could influence this parameter, perioperative complications and the outcomes in patients with inadequate LV remodeling.

## 2. Materials and Methods

All medical records of dogs affected by PDA arriving at the “Clinica Veterinaria Gran Sasso” (Italy) from January 2001 to December 2022 were retrospectively evaluated. 

All dogs affected by PDA were included in the study if their body weight at adult age was ≥15 kg and if they had at least one pre-interventional echocardiography and at minimum one post-interventional echocardiography after 24 h. To assess if the adult weight was more than 15 kg, the authors considered dogs of medium-large breeds and mongrel dogs that had a weight at presentation that was already >15 kg or that reached the goal weight at adult age confirmed after a telephone interview [19]. Exclusion criteria were the presence of other congenital/acquired cardiac or systemic disorders or a reversed PDA. Dogs with mitral valve regurgitation were included if the regurgitant jet was functional and not secondary to mitral valve morphological abnormalities.

In all selected patients, signalment, medical history, physical examination and body weight at presentation were registered. All the patients underwent interventional closure as soon as the diagnosis was made, therefore the age at presentation can be considered the age at ductal closure. Non-invasive systemic blood pressure (oscillometric method), dorsoventral and lateral thoracic radiographs were obtained, standard twelve-lead electrocardiograms were registered and a complete transthoracic echocardiography (TTE) was performed before and 24 h after PDA closure. The TTE exams were performed by cardiology residents or a cardiology diplomate (C.B.), and all exams were reviewed by an ECVIM board-certified cardiologist (C.B.). The bidimensional (B-mode), monodimensional (M-mode), spectral and color-flow Doppler images were obtained using Philips Epiq 7 [Philips Epiq 7C, Philips SpA Healthcare, Monza (MB), Italy], Esaote Mylab30Vet, Esaote Mylab60, Esaote Mylab X8, Esaote Mylab X90, [Esaote S.p.A., Firenze (FI), Italy], Mindray Vetus E7 [Mindray Medical Italy S.R.L. Trezzano sul Naviglio (MI), Italy] ultrasound machines, with a 1–5 array transducer, according to published recommendations [20].

The diagnosis of PDA was established when a vascular communication between the aorta and the main pulmonary artery (in the anatomic area of PDA) with left-to-right shunt was visualized through TTE. The measurement of MDD was obtained from a right parasternal short axis view. The M-mode measurements were obtained from the two-dimensional images of the largest transversal diameter of LV on right parasternal short axis view, at the level between the papillary muscles and the mitral valve. These measurements include the systolic and diastolic interventricular septum thickening, the systolic and diastolic LV posterior wall thickening and LV internal diameter at end-diastole (LVIDd) and end-systole (LVIDs), from which it is possible to calculate the endocardial shortening fraction (SF%) and ejection fraction (EF%) as an index of LV systolic function. The SF% was calculated by the following formula: [(LVIDd − LVIDs)/LVIDd] × 100 and the normal range value is 25.3–49.9 [21,22]. LV end-diastolic volume (EDV) and end-systolic volume (ESV) on M-mode modality were calculated using the Teichholz formula: EDV = (LVIDd^3^ × 7)/(LVIDd + 2.4) and ESV = (LVIDs^3^ × 7)/(LVIDs + 2.4). Values were indexed to the body surface area to obtain the end-diastolic volume index (EDVI), that has a normal value ≤ 100 mL/m^2^, and the end-systolic volume index (ESVI) with normal value ≤ 30 mL/m^2^ [10,23]. The EF% was calculated using the following formula: [(EDV − ESV) × 100]/EDV; the normal cutoff is >40–45% [24,25]. 

Any type of arrhythmia was recorded and evaluated though electrocardiographic examination. If any medical therapy was introduced before or after ductal closure it was recorded. 

All patients were re-checked with echocardiographic examination at the authors’ center 24 h after the procedure to identify the complete closure of the defect and to obtain measurements for evaluation of structural and functional heart changes. In all these dogs the previously described echocardiographic scans and measures were obtained. The echocardiographic evaluation after ductal closure allowed us to divide the patients into two groups based on the degree of heart remodeling after interventional procedure, compared to the echocardiography before closure: Group A included dogs with a reduction in ESVI after ductal closure compared to ESVI measured before the ductal closure; Group B included dogs who had no reduction in ESVI or had an increase in ESVI after closure. For the variables ESVI, SF%, EF% and body weight, each group was divided with a subclassification based on the age at presentation, dividing dogs in three categories: ≤6 months, >6 and ≤12 months, >12 months. Intraoperative or perioperative complications were recorded. 

Medical drugs administered before and after the interventional procedure were recorded. The difference between Group A and B were evaluated for every single administered drug and for the category of antiarrhythmic drug. 

In all dogs, a check-up was suggested at 1, 3, 6 and 12 months after PDA closure. Some of these dogs’ check-ups were performed at the authors’ center, for some of them the authors had follow-up by phone and the other patients were lost at follow-up.

The owners were contacted by phone for information about the outcome of the patients, and a telephone questionnaire was carried out. During the short interview they were asked if the patient was still alive, the general conditions of the dog and, in case of death, the causes. The causes of death were classified as “cardiac death” in case of congestive heart failure secondary to heart disease that brought the dog to die or sudden death for presumptive arrhythmias; all other causes of death were classified as “non-cardiac death”.

Statistical analysis was performed using IBM SPSS Statistics 27. Distribution of variables was tested for normality using the Shapiro–Wilk test at the α = 0.05 level. As centrally positioned values we considered the mean and the median with standard deviation (St.Dev) and interquartile range (IQR), their respective dispersion indices and confidence intervals (CI) were calculated at 95% confidence. Normally distributed data were compared using the two-sided Student’s t-test, for independent or paired samples as appropriate; non-normally distributed data were compared using the Mann–Whitney U-test for independent samples or Wilcoxon Signed Ranks Test for paired samples as appropriate; dichotomous variables were compared using the Chi-square test or Fisher’s exact test when appropriate. Categorical data were compared using the Chi-square test. Multiple comparisons were performed using one-way or two-way ANOVA with interaction and Tukey’s HSD *post hoc* tests. Correlation was tested with the Pearson correlation coefficient, with the following interpretation: ≤0.3 weak correlation, >0.3 and ≤0.7 moderate correlation, >0.7 strong correlation.

In order to assess the association of variables with a dichotomous response, logistic regression was performed using the backward method. An R square value greater than 0.1 at the 0.05 significance level was considered suitable.

A *p*-value of 0.05 was taken as statistical significance.

## 3. Results

Six hundred and three records of patients with PDA were analyzed, and one hundred fifty-seven dogs met the previously shown selection criteria. The dogs that were not included were excluded due to having a body weight inferior to 15 kg at adult age (390 dogs), because they were affected by associated congenital cardiopathies (33 dogs) or for the presentation of reversed PDA (23 dogs). 

At presentation, all included patients had increased dimension of LV in diastole and systole compared with the normal reference values previously described. 

Ninety-eight dogs were included in Group A (62.4%) and fifty-nine dogs were included in Group B (37.6%). Table 1 shows the distribution of breeds in Groups A and B, which suggests no significant difference related to breed between the two groups. In Group A the most evidenced breeds are German Shepherd (22.4%) and Mongrel (21.4%), followed by Border Collie (8.2%) and Dobermann (8.2%); in Group B the most common breeds are German Shepherd (30.5%), Mongrel (13.6%) and Dobermann (11.9%). 

The analysis of gender evidenced a higher number of females compared to males in both groups (respectively, Group A 81.6% and Group B 69.5%), but without statistically significant differences between groups. Considering the three age groups (equal or less than 6 months, between 6 and 12 months, older than 12 months), the distribution of dogs across the age groups is quite different in Groups A and B (see Table 2), although the difference is not statistically significant. In particular, in Group A the percentage of dogs younger than 6 months of age is more elevated compared to Group B (respectively, 40.6% and 29.3%), while dogs older than 12 months have a higher percentage in Group B (37.9%) compared to Group A (26.0%). 

In addition, the body weight was compared in the three age groups and a correlation between age and body weight emerged in dogs with age ≤ 6 months compared to other categories (*p*-value < 0.001).

**Figure 1:**
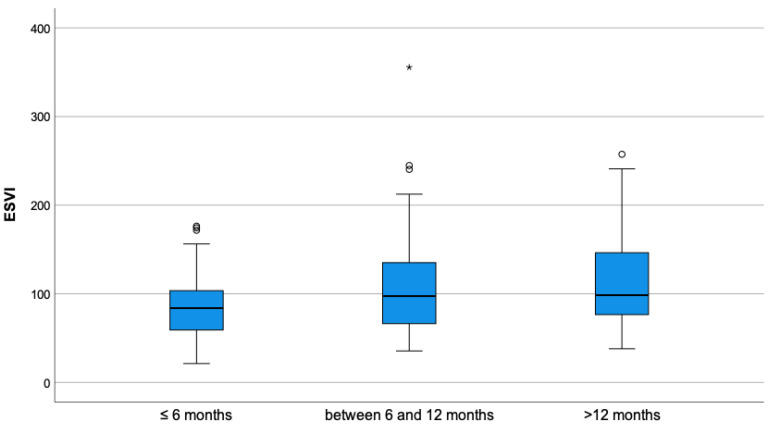
Boxplot of end-systolic volume index (ESVI) before ductal closure, stratified by age. A significant statistical difference was found between the group of dogs younger than 6 months and the group of dogs older than 12 months (*p*-value = 0.003). In the picture, as usual, the symbol “*” denotes an extreme value, which is far more than three times the interquartile range from the box, and the symbol “°” denotes an outlier, which is not an extreme value but is far more than 1.5 times the interquartile range from the box.

**Figure 2:**
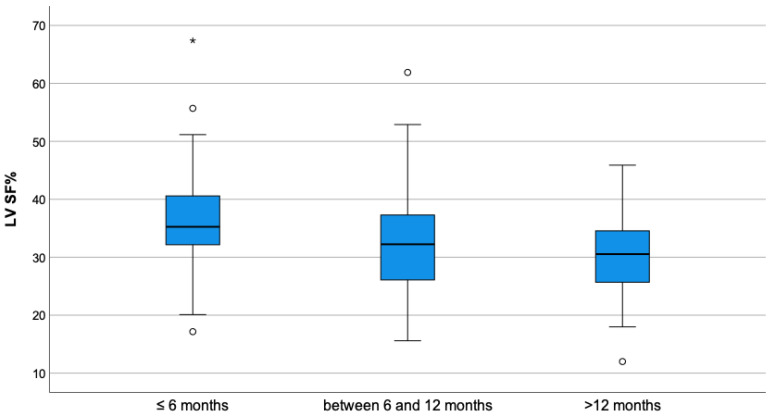
Boxplot of shortening fraction of left ventricle (LV SF%) before ductal closure, stratified by age. A significant statistical difference was found between the group of dogs younger than 6 months and all the other dogs (*p*-value < 0.001). In the picture, as usual, the symbol “*” denotes an extreme value, which is far more than three times the interquartile range from the box, and the symbol “°” denotes an outlier, which is not an extreme value but is far more than 1.5 times the interquartile range from the box.

**Figure 3:**
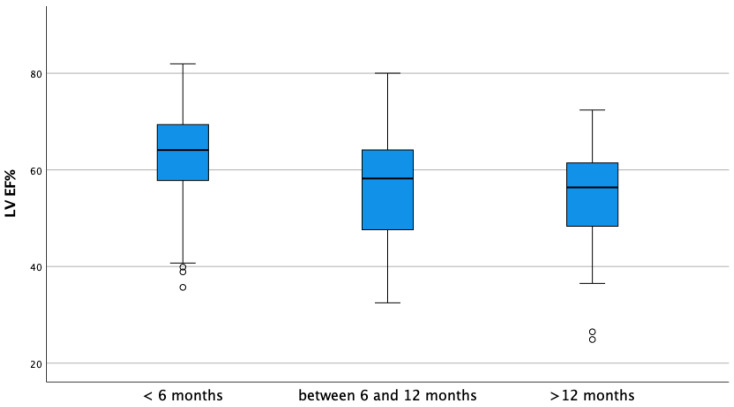
Boxplot of ejection fraction of left ventricle (LV EF%) before ductal closure, stratified by age. A significant statistical difference was found between the group of dogs younger than 6 months and all the other dogs (*p*-value < 0.001). In the picture, as usual, symbol “°” denotes an outlier, which is not an extreme value but is far more than 1.5 times the interquartile range from the box.

As shown in Table 3, which indicates the parameters before ductal closure, a statistically significant difference was observed in values of MDD (*p*-value = 0.023) and EDVI (*p*-value = 0.013) between groups, where Group B presented a higher value of all parameters compared to Group A. ESVI, SF% and EF% pre-closure were not statistically different between the two groups. The mean values of SF% and EF% before closure were within normal reference range in both groups. The age had a significant association with ESVI, in particular, the difference was clear between dogs less than 6 months old compared to dogs older than 12 months (*p*-value = 0.003), as shown in Figure 1. A similar association is seen for the SF% and EF% in different categories of age, in particular, in dogs younger than 6 months compared to the other categories (*p*-value < 0.001) (Figure 2 and Figure 3). 

Significant differences were found in body weight between the groups (*p*-value = 0.007); in Group A the mean value was 18.9 kg, compared to Group B where it was 22.6 kg. 

At presentation, in Group B, arrhythmias were present in 22.0% of dogs, while in Group A the dogs affected by arrhythmias were 5.1% of the total, and a statistical difference was evidenced (*p*-value = 0.001). The patients with arrhythmias presented atrial fibrillation (Group A: 5.1%; Group B: 15.2%), ectopic premature ventricular complexes single or organized in bigeminies (Group A: 0%; Group B: 6.8%) or an association of both (Group A: 0%; Group B: 3.4%). 

A PDA closure procedure was attempted in all dogs with a ACDO device. No dogs in Group A presented with complications during the procedure or in the following days, while 19 dogs (32.2%) in Group B presented with complications during interventional procedure or in the perioperative period, such as new onset arrhythmias (supraventricular tachycardia in two dogs, isolated or organized premature ventricular complex in six dogs, paroxysmal ventricular tachycardia in three dogs) or pulmonary oedema (eight dogs). 

In all dogs, a complete ductal closure without residual flow was observed 24 h after the interventional procedure. 

**Figure 4:**
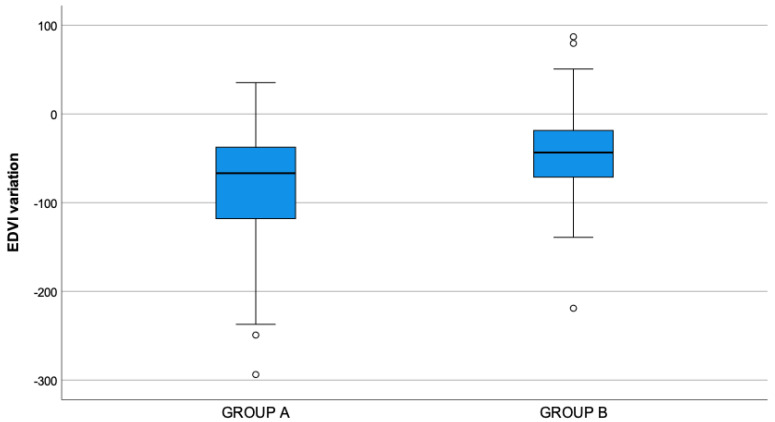
Variation in end-diastolic volume index (EDVI) values in group A and B, calculated as the difference between the post-closure and pre-closure values (negative values correspond to a reduction in EDVI, while positive values correspond to an increase). Statistically significant (*p*-value < 0.001) greater reduction was found in group A with respect to group B. In the picture, as usual, symbol “°” denotes an outlier, which is not an extreme value but is far more than 1.5 times the interquartile range from the box.

On post-closure echocardiography, a significant reduction in left ventricular diastolic dimension, calculated with EDVI, was observed in both groups (*p*-value *=* 0.001). The comparison between Groups B and A showed a greater statistically significant (*p*-value < 0.001) reduction in the left ventricular diastolic dimension in the latter, as shown in Figure 4. In particular, in Group A the mean reduction was 81.9 mL/m^2^ (95% CI: 69.7–94.2), and in Group B the mean reduction was 43.1 mL/m^2^ (95% CI: 28.9–57.3). From analysis of the post-closure LV dimension in systole, in Group A there was a significant reduction in ESVI compared to the value before closure (*p*-value = 0.001), and the mean values were, respectively, 96.5 mL/m^2^ and 76.9 mL/m^2^ (Table 3 and Table 4). In Group B there was a significant increase in ESVI compared to the value before surgery (*p*-value = 0.001); mean values were, respectively, 111.2 mL/m^2^ and 139.2 mL/m^2^ (Table 3 and Table 4). Moreover, the comparison between groups showed a higher statistically significant value of ESVI in Group B compared to those of Group A in post closure echocardiography (*p*-value < 0.001) (Table 4). 

The SF% presented a significant reduction after ductal closure compared to pre-closure in both categories (*p*-value < 0.001 in both cases), and between the groups there was a significant difference between the values of SF% after ductal closure with a higher reduction in the value in Group B, as shown in Table 4 (21.3% vs. 27.7%). Moreover, in Group B the mean value of SF% was below the normal reference range, whilst in Group A the value was within the normal reference range indicated previously. 

The same behavior was shown regarding EF%, where a significant reduction after ductal closure compared to pre-closure in each group was evidenced (*p*-value < 0.001 in both cases), and comparing the mean value of EF% after ductal closure in Groups A and B there was a significantly larger reduction in Group B, as evidenced Table 4.

After ductal closure, dogs in Group B presented with more arrhythmias compared to Group A (*p*-value = 0.001).

Medical drugs administered before the interventional procedure included Furosemide (Group A 34.3%–Group B 47.5%), ACE-inhibitors (Group A 33.3%–Group B 47.5%), Pimobendan (Group A 15.2%–Group B 20.3%), Spironolattone (Group A 1%–Group B no dogs), Digoxin (Group A 1%–Group B 3.4%), a mixture of Digoxin and Diltiazem (Group A no dogs–Group B 5.1%), Sotalol (Group A 2%–Group B 5,1%), and Amiodarone (Group A 1%–Group B 11.9%) or a mixture of Digoxin and Amiodaron (1% in Group A–Group B no dogs). The category of antiarrhythmic drugs, which included Digoxin, Diltiazem, Sotalol and Amiodarone, presented a significatively higher administration in Group B (*p*-value < 0.001) compared to Group A. Analyzing differences for single drug, only Amiodarone was significantly higher in Group B (*p*-value = 0.027), all the others did not present any difference between the groups. 

There was not a significant difference between groups for the assumption of medical therapy before and after ductal closure; however, medical therapy after ductal closure was prescribed/maintained in 44.1% of dogs in Group B and 38.8% in Group A. 

At the time of writing, information about the outcome was available for in 55 dogs from Group A (55.5%) and 39 dogs from Group B (66.1%): forty-six (83.6%) dogs from Group A and twenty-two (56.4%) dogs from Group B were alive, while nine dogs (16.4%) from Group A and seventeen dogs (43.5%) from Group B were dead.

The cause of death was categorized as cardiac or non-cardiac: in Group A two (22%) of the dead dogs died of cardiac death, compared to thirteen (76%) of the dead dogs from Group B, with statistically significant difference (*p*-value = 0.01). 

## 4. Discussion

In this study the authors analyzed, using TTE, the short-term structural and functional changes in the LVs in medium and large sized dogs before and after ductal closure, the perioperative complications and the outcomes.

In all patients in this work, a dilation of the LV was evidenced at presentation, in accordance with previous studies in veterinary and human medicine [4,10,26,27,28]. This is caused by the presence of an extracardiac left-to-right shunt with continuous passage of flow through the defect, which induced a volume overload in pulmonary circulation, and consequently a volume overload in the left-side of the heart with primary dilation of the LV and subsequently the left atrium [4,6,29]. After ductal closure, changes in the hemodynamic status are seen, in particular the interruption of the shunt flow induced an increased the flow in systemic circulation with a consequently increased afterload, associated with normalization of pulmonary flow and reduced preload, this provokes a reduction in the dimension of the LV and a reduced stretch of the ventricular wall [4,9,30]. Sometimes this process, defined as “afterload mismatch”, can cause systolic dysfunction of the left ventricle that can be indicated by a decrease in SF% and EF% in dogs and humans [13,31,32]. However, the SF% and EF% cannot be considered the gold standard for the evaluation of systolic function in dogs, the speckle-tracking echocardiography may be a more appropriate tool to assess cardiac contractility in dogs with PDA [33]. In particular, in dogs that underwent PDA closure, it is possible to show a reduction in SF% and EF% and a reduction in parameters indicative of circumferential contractility (as circumferential, radial, and transversal Strain and Strain-Rate), but a maintenance of long axis contractility indicated by longitudinal Strain and Strain Rate [24].

In humans, a different response to LV remodeling and systolic function can be seen after ductal closure, based on the dimension of ductal diameter and the age of the patients [9,27]. This is also reported in dogs [4,16]. In adult humans, a post-closure deterioration of the LV is suspected when an immediate decrease in LV EDVI is evidenced, associated with a stationary ESVI value [27]. The patients in this study were divided into two groups based on the degree of heart remodeling required 24 h after ductal closure: Group A included patient with a physiologic heart remodeling, with a reduction in EDVI and ESVI after closure, while Group B included all dogs without ESVI reduction after closure. 

The results of this work show that the different response of the LV after ductal closure in dogs is influenced by body weight, dimension of MDD, diastolic volume of LV and arrhythmias at presentation. Breed and gender do not have an influence on post-closure remodeling; however, from the statistical analysis it emerged that the most commonly affected gender was female, as previously reported in reference [1]. The most commonly recorded breeds in both groups were German Shepherd, Mongrel and Dobermann, and also Border Collie in Group A; these breeds were previously indicated in other studies [1,2]. 

The echocardiographic evaluation showed that the LV dimension in diastole before closure was more elevated in Group B compared to Group A; this could be secondary to the larger dimension of MDD also evidenced in Group B, which causes a greater quantity of flow to pass through the defect and may have an impact on the degree of the increasing preload, as previously reported in humans and dogs [4,6,9]. In all analyzed dogs there was a statistically significant reduction in LV dimension in diastole after PDA closure secondary to the reduction of preload, as expected from the literature, but this study evidenced that the reduction is significantly greater in dogs with physiologic heart remodeling, defined as Group A [4,9,30]. The authors believe this may be due to a minor dilation of the left ventricle before ductal closure, which induced a minor stretch in ventricular muscle and a better heart recovery. 

In our study, before PDA closure, when following the physiopathological mechanism, there is a condition of high preload and low afterload, the mean value of SF% and EF% were within normal range and there was an absence of statistical difference in these parameters between the groups. After closure, where on physiopathological bases there is an increased afterload and a reduced preload, in our study a significative reduction in SF% and EF% in both groups was evidenced, as previously demonstrated in references [13,24,31]. Although, the degree of reduction was different, in Group B the SF% and EF% were lower, the value of FS% was below the normal reference range, and the EF% was near to the lower cutoff value, while in Group A, with physiologic heart remodeling, both values were within the normal reference range [21,22,25,33]. Both the SF% and EF% could be used as indicators of systolic function in dogs with some limitations, but an important aspect for the authors is that these parameters measured from M-mode could be used also as an indicator of parietal stress in the left ventricle, following Laplace’s law, because they derive from the difference between the diastolic and systolic diameter of the chamber measured in its larger portion, as previously reported in human and veterinary literature [10,24,27]. This aspect is less evaluable with a bidimensional view of left ventricle. The M-mode measurement of SF% and EF% as an index of systolic function has limitations because they could be affected by the hemodynamic state of the patient, by preload, afterload, heart rate, arrhythmias or severe mitral insufficiency, and FS% very often overestimates the size of the LV [22,34,35]. Although usually the evaluation of EF% is performed with bidimensional view of left ventricle, the measure in monodimensional view, as performed in this study, is reported in premature infants and shows a good concordance with the bidimensional view in dogs [14,24,33]. The reduction in SF% and EF% in Group B could indicate a worsening of systolic function in the left ventricle [22,32], although this could also be a consequence of the severe enlargement of the left ventricle before the duct closure. In fact, in Group B, in which the left ventricle was more severely stretched, there could have been a greater parietal stress which might have been evident after the duct closure because of the physiological mechanisms previously mentioned. This may have determined an increase in the left ventricle dimensions in the end-systolic phase, which inevitably reduced the SF% and EF% values [24,27]. This could also be in concordance with the fact that the mean EF% in our patients was not under the reference range. Despite the limits of M-mode, the authors highlight that in patients with a so severe a left ventricular enlargement, like dogs with PDA, a risk of difficult alignment to the left apical view used for B-mode measurement with a potential underestimation of the values is possible. Furthermore, it is interesting to note, as Scollan et al. described, a good agreement between the mono-dimensional, bi-dimensional, three-dimensional and Computed Tomography measurement of left ventricular volume in healthy dogs, while in human medicine a correlation between three-dimensional and Computed Tomography measurement of left ventricular volume has been shown [36].

Dogs with insufficient LV systolic remodeling after PDA closure had a higher body weight at presentation: it is possible to hypothesize that, in larger sized dogs the severe overload of LV induces a decreased adaptation of parietal wall to stress, deriving from the increase in the LV transversal diameter, according to Laplace’s law, as confirmed by previous studies [10,18]. The possible explanation of the association between reduced left systolic heart remodeling and a possible reduced systolic function is that a larger LV dimension induces excessive parietal distension and a severe stretch of sarcomeres. This could cause difficult interactions between actine filaments and cross-bridge of myosin, inducing a minor capacity of contractility of the ventricular chamber, so this may indicate that a very large PDA may weaken the LV before closure; however, this aspect often becomes evident after PDA closure for the onset of increased afterload [8,30,37]. 

In this study, the body weight at presentation was influenced by age, in particular there was a correlation between the age and body weight in dogs with age ≤ 6 months. The analysis of medium and large size dogs could have influenced this aspect because the growing curve is greater in that period of life compared to dogs aged over 6 or 12 months. 

In human medicine age influences the post-closure outcome of the patient, in particular the study of Jeong et al., conducted in adults with relatively large PDA size, evidenced an increased deterioration in systolic function compared to children; this was attributed to the prolonged LV remodeling induced by PDA in adults compared to children which might cause some structural changes in the LV myocardium and irreversible deterioration of LV contractility [9,27]. In accordance with some veterinary literature, in this paper the age is not a significative different parameter between groups; however, in this study the higher percentage of young dogs in Group A and the higher number of dogs older than one year of age in Group B could be statistically significant considering a larger sample size, indicating, as reported by other authors in other studies, a worsening of left ventricle dilation over time and consequent myocardial deterioration [16,17,18]. Furthermore, the smaller ESVI and the greater SF% and EF% in younger dogs with PDA compared to older dogs may confirm better plasticity, capacity of adaptation and *restitutio ad integrum* of younger hearts, but unfortunately, the hemodynamic condition evidenced with ductal patency makes it impossible to predict patients who will develop complications based on values such as ESVI or SF% before closure. In dogs with reduced remodeling of the heart after ductal closure, there is a higher risk of developing perioperative complications, like pulmonic edema or arrhythmias, or death due to cardiac causes compared to other dogs. The pulmonic edema may be due to a more severe left ventricle dilation at presentation and a worse cardiac performance after ductal closure in more stretched hearts. The severe dilation of the left ventricular chamber can induce a reduced compliance of left ventricle with higher end-diastolic pressure. A mismatch between the left ventricle and the Aorta, originated from the PDA closure could be also present, and this increases the residual volume of the left ventricle, inducing an even greater reduction in ventricular compliance. All these consequences could induce pulmonary edema. These conditions are often associated with the presence of larger MDD, with severe left heart enlargement and arrhythmias, that could determine an increased risk of sudden death or congestive heart failure with consequent death. In this study, the pulmonic edema, as a manifestation of congestive heart failure, was reported more than in other studies; this could be due to the fact that the analyzed population includes only medium and large-size dogs, which are less able to tolerate severe heart enlargement compared to small breed dogs [4,10,18,32]. As previously reported, an earlier ductal closure in medium and large size dogs is indicated when possible; however, even if patients are presented at older age, the PDA closure must be performed because the untreated persistence of the shunt can induce congestive heart failure, arrhythmias or sudden death [7,10]. 

The presence of arrhythmias before PDA closure, or which emerged in the perioperative period, can be considered as a possible factor which predisposes patients to abnormal heart remodeling after closure. The arrhythmias highlighted are atrial fibrillation and premature ventricular ectopic complex, which could be secondary to severe left atrial and ventricular stretch respectively [38,39]. The hypothesis of the authors is that a more severe heart enlargement, as evidenced in Group B, may justify an increased risk of developing arrhythmias, but also that arrhythmias could worsen the degree of left ventricle remodeling and potentially influence systolic function.

Relative to medical therapy, the absence of statistical difference between the two groups regarding the use of drugs such as Furosemide, ACE-i, Pimobendan and Spironolactone could be due to the fact that dogs in both groups presented significant cardiac remodeling at presentation, as previously reported. Antiarrhythmic drugs were prescribed most in Group B compared to Group A, because Group B was more affected by arrhythmias than Group A at presentation. Amiodarone is the only drug which was prescribed more in Group B compared to Group A at presentation. This drug seems to have a scarce impact on systolic function in dogs, therefore the differences in heart remodeling after PDA closure between the groups cannot be attributed to the pharmacodynamics of this therapy [40]. In human medicine, the effect of amiodarone on heart contractility is debated. In some studies there is an improvement in systolic function or cardiac output, while in others the intravenous administration of this drug depresses myocardial contractility in isolated hearts [41,42,43]. 

Comparing the groups, there was not a statistically significant difference regarding the administration of medical therapy both before and after closure, this aspect could be secondary to the fact that medium and large dogs have a worse adaptation of the parietal wall to stress, therefore they often need medical therapy before and immediately after closure of the PDA [10,18]. However, in Group B, the percentage of dogs that took medical therapy was higher; this might bring out a significance in an enlarged sample, in the future. 

The study presents some limitations. The main ones are related to the monodimensional measurement of the LV and the evaluation of systolic function only with SF% and EF%. The dimensions of the LV were measured with the Teichholz equation in M-mode, without evaluating the complex geometry of the left ventricle; however, this method is the most repeatable so it is easy to check during the follow-up of the same patient, it measures the largest diameter of left ventricle in diastole which indicates the point of maximum parietal stress and Spalla et al. found a good correlation among the Teichholz method, the area-length method and allometric scaling, regardless of the different underlying geometrical assumption [10,33]. The SF% and EF% are an easy way to measure the systolic function in dogs and they are one of the most frequently used indices, although but they have some limitations: they are affected by the angle between the cursor and the left ventricular walls (for a correct measurement this angle must be as close as possible to 90 degrees) and they could be affected by the hemodynamic condition of the patient, such as variations in preload, afterload or heart rate, the presence of arrhythmias or severe mitral insufficiency. Furthermore, above all these parameters measure radial contractility [22,35]. Due to the retrospective nature of the study, a global assessment of LV systolic function and dimensions using two-dimensional Simpson methods, the Strain or the Strain Rate evaluation was not possible. In this group of dogs the measurement of blood level of Taurine and Carnitine as possible factors influencing systolic function was not performed; however, in their medical history, no patients assumed diets that could be a predisposing factor to low levels of these parameters, such as diets containing legumes, grain-free, novel protein sources or home diet [35,44,45]. The evaluation of MDD in this study was performed predominantly through right parasternal view. From 2007, some studies evidenced that the best transthoracic way to evaluate the MDD is through the left cranial view [46,47,48]. Due to the retrospective analysis of this work, and the inclusion of a lot of cases in the period before of 2007, the authors decided to include the measurement in right parasternal view because it was registered in all patients. Moreover, currently the authors also measure the MDD with the right parasternal view because sometimes the MDD is better visualized with this view, according to the thorax morphology of different breeds. Another limitation is the analysis of a single follow-up echocardiography 24 h after ductal closure. 

A proposed future study may be a prospective study with a higher number of medium and large size patients, where more accurate echocardiographic parameters for evaluation of global systolic function (as bi-dimensional Simpson method with measurement of EF%, tissue Doppler, Strain and Strain-Rate) will be used, with the aim of identifying pre-closure echocardiographic predicting values for the risk stratification of the dogs for the development of complications after PDA closure. Moreover, it will be interesting to study the degree and the duration of long-term heart remodeling and function with echocardiographic follow-up performed over time.

## 5. Conclusions

The results of this study show that dogs with a higher body weight, a ductus with larger MDD, a more severe heart enlargement or arrhythmias at presentation have an increased risk of developing worsening structural and functional condition after ductal closure, which may be associated with perioperative complications and cardiac death. Furthermore, the systolic dimensions of the left ventricle, SF% and EF% were influenced by age, as evidenced in dogs younger than 6 months which had a minor dilation of the left ventricle in systole, less parietal stretch and a better systolic function. Therefore, when possible, the therapeutic indication is to perform PDA closure as early as possible, in younger dogs with less heart remodeling and major functional capacity; however, even if patients are presented at older age, the PDA closure must be performed because the untreated persistence of the shunt can induce complications such as congestive heart failure, arrhythmias or sudden death. 

## Figures and Tables

**Table 1:** Distribution of breeds in Groups A and B.

	Group A		Group B		Total	
	n.	perc.	n.	perc.	n.	perc.
Akita Inu	0	0.0%	1	1.7%	1	0.6%
Australian Shepherd	7	7.1%	2	3.4%	9	5.7%
Belgian Shepherd	4	4.1%	1	1.7%	5	3.2%
Bernese Mountain dog	6	6.1%	3	5.1%	9	5.7%
Bobtail	0	0.0%	1	1.7%	1	0.6%
Border Collie	8	8.2%	2	3.4%	10	6.4%
Dobermann	8	8.2%	7	11.9%	15	9.6%
German Shepherd	22	22.4%	18	30.5%	40	25.5%
Greyhound	1	1.0%	1	1.7%	2	1.3%
Hunting dog	3	3.1%	1	1.7%	4	2.5%
Ibizan Hound	0	0.0%	1	1.7%	1	0.6%
Maremmano Sheepdog	0	0.0%	2	3.4%	2	1.3%
Mongrel	21	21.4%	8	13.6%	29	18.5%
Neapolitan Mastiff	0	0.0%	1	1.7%	1	0.6%
Newfoundland	5	5.1%	1	1.7%	6	3.8%
Pitbull Terrier	2	2.0%	0	0.0%	2	1.3%
Retriever dog	5	5.1%	2	3.4%	7	4.5%
Rottweiler	1	1.0%	0	0.0%	1	0.6%
Setter dog	3	3.1%	3	5.1%	6	3.8%
Swiss Shepherd	2	2.0%	0	0.0%	2	1.3%
Weimaraner	0	0.0%	4	6.8%	4	2.5%
TOTAL	98	100.0%	59	100.0%	157	100.0%

**Table 2:** Distribution of dogs categorized by age between Groups A and B.

	Group A		Group B		Total	
	n.	%	n.	%	n.	%
≤6 months	39	40.6	17	29.3	56	36.4
>6 and ≤12 months	32	33.3	19	32.8	51	33.1
>12 months	25	26.0	22	37.9	47	30.5
TOTAL	96	100	58	100	154	100

**Table 3:** Echocardiographic parameters before ductal closure: MDD TTE (minimal ductal diameter evaluated with transthoracic echocardiography) measured in mm, EDVI MMODE (End-diastolic volume index in monodimensional view) and ESVI MMODE (End-systolic volume index in monodimensional view) measured in mL/m^2^, shortening fraction of left ventricle (LV SF), ejection fraction of left ventricle (LV EF%), expressed as a percentage.

	Group A				Group B				
	Mean	Median	Standard Deviation	IQR	Mean	Median	Standard Deviation	IQR	*p*-Value
MDD TTE (mm)	5.6	5.0	2.3	1.9	6.41	5.9	2.23	2.1	0.023
EDVI MMODE (mL/m^2^)	235.1	218.3	86.0	107.7	274.5	268.8	107.4	144.8	0.013
ESVI MMODE (mL/m^2^)	96.5	85.1	46.2	51.9	111.2	101.1	61.8	62.2	not significative
LV SF (%)	33.07	32.5	8.36	9.3	33.8	34.1	8.7	10.0	not significative
LV EF (%)	57.9	59.5	11.2	14.0	59.8	61.7	11.2	15.5	not significative

**Table 4:** Echocardiographic parameters after ductal closure. MDD TTE (minimal ductal diameter evaluated with transthoracic echocardiography) measured in mm, EDVI MMODE (End-diastolic volume index in monodimensional view) and ESVI MMODE (End-systolic volume index in monodimensional view) measured in mL/m^2^, shortening fraction of left ventricle (LV SF), ejection fraction of left ventricle (LV EF%), expressed as a percentual.

	Group A				Group B				
	Mean	Median	Standard Deviation	IQR	Mean	Median	Standard Deviation	IQR	*p*-Value
EDVI MMODE (mL/m^2^)	153.2	143.4	58.1	62.4	230.1	225.9	101.5	126.9	<0.001
ESVI MMODE (mL/m^2^)	76.9	66.2	37.9	45.7	139.2	128.7	71.5	82.5	<0.001
LV SF (%)	27.7	26.5	8.9	11.9	21.3	20.0	8.41	10.55	<0.001
LV EF (%)	49.1	49.4	14.0	17.9	41.1	39.6	12.7	18.0	<0.001

## Data Availability

Data are contained within the article.
